# Changes in Permittivity of the Piezoelectric Material PVDF as Functions of the Electrical Field and Temperature

**DOI:** 10.3390/ma14195736

**Published:** 2021-10-01

**Authors:** You Zhou, Mona Zaghloul

**Affiliations:** School of Engineering and Applied Science, The George Washington University, Washington, DC 20052, USA

**Keywords:** Polyvinylidene Fluoride, PVDF, permittivity, piezoelectric material, COMSOL simulation

## Abstract

Polyvinylidene Fluoride (PVDF) is becoming a widely used piezoelectric material because of its flexibility, low cost, light weight, and biocompatibility. Its electronic properties, such as its permittivity, can be affected by material crystal structure variations, which also greatly impact the material’s application. It is known that external stress and electrical fields can alter the crystal structure of piezoelectric material. In this research, we aim to investigate the relationship between the external electrical field and the permittivity property of PVDF material. The basic standard equations, finite element analysis, and experimental measurement are included in this paper. By using sweeping voltages from −25 V to +25 V using an Agilent Technologies B1500A Semiconductor Device Analyzer, an increase in the permittivity of the PVDF material is observed. In this work, the study of the permittivity change under the application of different electrical fields at room temperature is presented, and the application of the electrical field under different temperatures is also studied and presented.

## 1. Introduction

The piezoelectric effect was first discovered by Pierre Curie and Jacques in the 1880s [1]. This effect describes the conversion of mechanical energy into electrical energy and vice versa [2,3]. Piezoelectric materials are available in natural form and can also be artificially synthesized. Quartz, Rochelle salt, and tourmaline are examples of natural piezoelectric materials. Some piezoelectric materials, such as Lead Zirconium Titanate (PZT), Lead Lanthanum Zirconate Titanate (PLZT), Aluminum Nitride (ALN), Zinc Oxide (ZnO), and Polyvinylidene Fluoride (PVDF) are artificially synthesized [3,4]. The piezoelectric property is related to the material’s non-centrosymmetric crystal structure [5]. Polarization happens when an external force is applied to the material and the electrical potential is exhibited within the material at the same time [6].

In addition to the external force case, the electrical field can also affect the crystal structure of the piezoelectric material, which also leads to a change in permittivity [3]. An increase in the value of the permittivity of PZT-5H was reported following the application of an AC bias [7]. This is critical for applications of piezoelectrical material because the influence of dielectric constant variations on the piezoelectric material is not neglectable. No matter whether the material is used as a sensor or as an actuator, the performance of the piezoelectric material changes if an external electrical field can change the material’s properties. However, if the dielectric constant of the material can be controlled precisely, the unstable performance problem can be solved. 

PVDF is a commonly used piezoelectric material because of its good flexibility, light weight, low cost, and biocompatibility, and it has the potential to be used in many biomedical applications [8,9,10]. For example, PVDF, a flexible piezoelectric material can be used to measure cardiorespiratory signals [11,12,13]. PVDF cable sensors are commercially available to detect thorax displacement by respiratory and ballistic movement by heartbeat [14,15]. The cable is a capacitor made of a long single PVDF film, outer conductive braid shield, and inner copper center core. A charge is generated between the center core and the outer braid shield when the cable is stretched or compressed by the movement [16]. However, this charge is usually very small and can be easily affected. Even small capacitance variations can affect the measurement result. When the temperature changes or an external electrical field is applied to the material, the permittivity will change, which results in a capacitance change. This change will lead to an unexpected charge change that may affect the measurement results. Since these kinds of biomedical devices usually work at body temperature or room temperature, which do not change a lot, this work mainly focuses on the effect of the electrical field on the permittivity of PVDF. The permittivity of PVDF can be changed by mixing it with BaTiO3 [17]. In this work, we consider the characterization of the permittivity of PVDF under the influence of an external electric field. We study the piezoelectric property and the permittivity of the PVDF material. By using a COMSOL simulation and experiment measurements, we measure the capacitance versus the applied voltage and calculate the permittivity change under the application of an electrical field at room temperature and different temperatures.

## 2. Materials and Methods

### 2.1. Basic Standard Equations for Piezoelectric Materials

Strain–Charge and Stress–Charge equations are usually used to express the piezoelectric effect. Most material data show the Strain–Charge form instead of the Stress–Charge form. However, data in Strain–Charge form can be transformed into Stress–Charge form for the variation formulation used by the COMSOL simulator for discretization and computation [18]. In this paper, we only show the Strain–Charge form equations to explain the piezoelectric effect. 

#### 2.1.1. Strain-Charge Form

Equations (1) and (2) express the piezoelectric effect in the Strain–Charge form.

(1) D=dT+εE

(2) $$S=s_{E}T+d^{T}E\;$$

where *S*, *D*, *T*, and *E* are the strain, electric displacement, external mechanical stress, and electric field in Equation (1), respectively. The material parameters $s_{E}$, ε, and *d* are material compliance, permittivity, and piezoelectric coefficients, respectively [12]. From these two equations, we know that when no external stress is applied to a piezoelectric material, the strain mainly depends on the external electrical field. To illustrate the directions of the piezoelectric effect, matrix forms of Equations (1) and (2) are expressed as Equations (3) and (4).

(3) $$\left[\begin{array}{c} D_{1}\\ D_{2}\\ D_{3} \end{array}\right]=\left[\begin{array}{ccc} d_{11} & \cdots & d_{16}\\ \vdots & \ddots & \vdots \\ d_{31} & \cdots & d_{36} \end{array}\right]\left[\begin{array}{c} T_{1}\\ T_{2}\\ T_{3}\\ T_{4}\\ T_{5}\\ T_{6} \end{array}\right]+\left[\begin{array}{ccc} \varepsilon_{11} & \varepsilon_{12} & \varepsilon_{13}\\ \varepsilon_{21} & \varepsilon_{22} & \varepsilon_{23}\\ \varepsilon_{31} & \varepsilon_{32} & \varepsilon_{33} \end{array}\right]\left[\begin{array}{c} E_{1}\\ E_{2}\\ E_{3} \end{array}\right]$$

(4) $$\left[\begin{array}{c} S_{1}\\ S_{2}\\ S_{3}\\ S_{4}\\ S_{5}\\ S_{6} \end{array}\right]=\left[\begin{array}{ccc} s_{E}{}_{11} & \cdots & s_{E}{}_{16}\\ \vdots & \ddots & \vdots \\ s_{E}{}_{61} & \cdots & s_{E}{}_{66} \end{array}\right]\left[\begin{array}{c} T_{1}\\ T_{2}\\ T_{3}\\ T_{4}\\ T_{5}\\ T_{6} \end{array}\right]+\left[\begin{array}{ccc} d_{11} & \cdots & d_{31}\\ \vdots & \ddots & \vdots \\ d_{16} & \cdots & d_{36} \end{array}\right]\left[\begin{array}{c} E_{1}\\ E_{2}\\ E_{3} \end{array}\right]$$

where subscripts 1, 2, and 3 are used to define the directions along the X, Y, and Z axes, respectively. Subscripts 4, 5, and 6 show the shear directions of the X, Y, and Z axes, respectively.

#### 2.1.2. Permittivity Calculation

**Figure 1:**
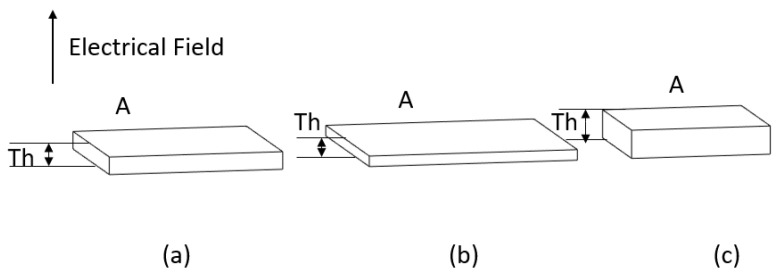
Illustration of the PVDF response to an applied electrical field (**a**) piezoelectric PVDF under no electrical field. (**b**) PVDF sheet under an electrical field with the opposite direction to the polling direction (**c**) PVDF sheet under electrical field with the same direction as the polling direction.

Consider a rectangular PVDF sheet with surface area A and thickness Th, as shown in Figure 1. When applying an electrical field with the same direction as the polling direction of the piezoelectric material, the thickness increases and the area decreases. On the other hand, when applying an electrical field with the opposite direction to the polling direction of the piezoelectrical material, the thickness decreases while the area increases [19]. 

**Figure 2:**
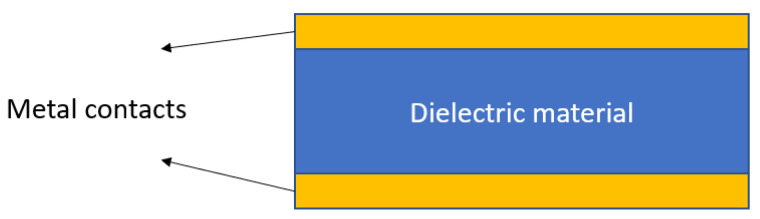
Illustration of a capacitor used for the measurement of permittivity.

Permittivity, which is also called the dielectric constant, refers to the ability of a material to store electrical energy under the influence of an electrical field. Figure 2 shows a capacitor with double-sided metal contacts used to apply an electrical field and a dielectric material between the metal contacts.

Permittivity can be determined from the measurement of capacitance, area, and thickness under an electric field, according to Equation (5).

(5) C=εA/Th

*C*, ε, A, and Th are the capacitance, permittivity, surface area, and thickness, respectively. ε = $\varepsilon_{r}\times \varepsilon_{0}$, where $\varepsilon_{0}$ is the vacuum permittivity and $\varepsilon_{r}$ is the relative permittivity or dielectric constant of the material. Due to the non-centrosymmetric crystal structure effect, the applied electric field will change the thickness and surface area as well as the permittivity of the PVDF sheet.

### 2.2. Material Properties

The material used in this work was PVDF. It was provided by Measurement Specialties, Inc., a TE Connectivity Company [20]. The material used was a metalized PVDF film sheet with electrodes on both sides with part number 1-1004347-0. The surface area of the overlapping electrodes was 0.02413 m^2^ while the thickness was 28 um, according to the datasheet. Since we were interested in the permittivity change under an electrical field rather than the permittivity under no electrical field, the small difference between the real value and value on the datasheet would not affect the results. 

The piezoelectric constant *d*_33_ was −33 × 10^−12^ m/V and the density ρ was 1780 kg/m^3^. The elastic compliance $s_{E}$ was

(6) $$\begin{array}{l} s_{E}=\left[\begin{array}{ccc} s_{E}{}_{11} & \cdots & s_{E}{}_{16}\\ \vdots & \ddots & \vdots \\ s_{E}{}_{61} & \cdots & s_{E}{}_{66} \end{array}\right]\\ =\left[\begin{array}{cccccc} 3.781 & -1.482 & -1.724 & 0 & 0 & 0\\ -1.482 & 3.781 & -1.724 & 0 & 0 & 0\\ -1.724 & -1.724 & 10.92 & 0 & 0 & 0\\ 0 & 0 & 0 & 14.28 & 0 & 0\\ 0 & 0 & 0 & 0 & 11.1 & 0\\ 0 & 0 & 0 & 0 & 0 & 11.1 \end{array}\right]\;\times \;10^{-10}(1/\mathrm{Pa})\; \end{array}$$

The piezoelectric coefficient $d_{ij}$ was

(7) $$d_{ij}=\left[\begin{array}{cccccc} 0 & 0 & 0 & 0 & 0 & 0\\ 0 & 0 & 0 & 0 & 0 & 0\\ 23 & 1.476 & -33 & 0 & 0 & 0 \end{array}\right]\left[pC/N\right]$$

*d*_31_ and *d*_33_ were 23 pC/N and −33 pC/N, respectively. They were obtained from the datasheet. *d*_32_ was 1.476 pC/N, as determined with COMSOL. 

### 2.3. Simulation in COMSOL 5.6

**Figure 3:**
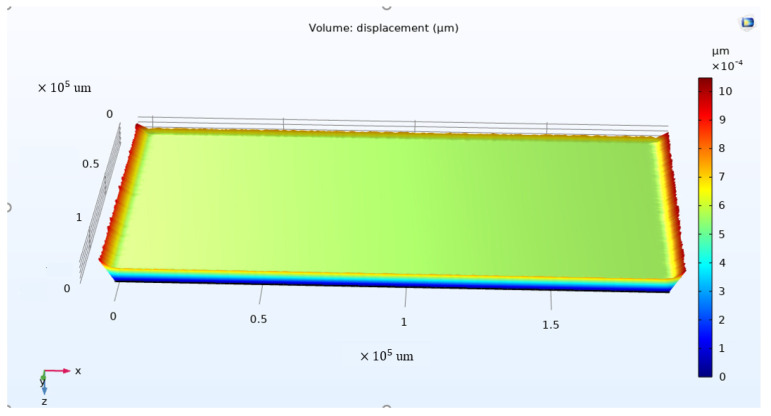
Simulation of thickness variation when applying 25 V to the PVDF.

To calculate the permittivity, the values of thickness, area, and capacitance were needed. However, the thickness change under the electrical field could not be directly measured because the material was too thin. Thus, a first simple estimation of the variation in the thickness was done by multiplying the *d*_33_ parameter by the electrical field and further multiplying it by the thickness of the PVDF sheet. The applied voltage was divided by the thickness of the material into electrical fields between the top and bottom surfaces of the PVDF sheet. The calculation shows that the estimated thickness change was about 0.8 nm, a small and negligible amount for the capacitance analysis when applying 25 V. To verify the estimation, a COMSOL 5.6 simulation was used to determine the change in thickness under the applied electrical voltage. The advantage of COMSOL is the ease of setting up a multi-physics simulation. As it uses one pre-processor, solver, and post-processor, simulation analysts do not have to use a wide range of tools. In this work, we used piezoelectric effect Multiphysics in the COMSOL simulator, which includes Solid Mechanic Physics and Electrostatic Physics. The dimensions of the model assigned in the simulation were the same as what we used in the experiment. The area of the PVDF sheet was 0.02413 m^2^, while the thickness was 28 um. Under different applied voltages, the changes in the area and thickness were different. For example, if 25 V was applied to the material (the range for the Agilent Technologies B1500A Semiconductor Device used in the laboratory), a thickness change of 0.6 nm occurred, as illustrated in Figure 3. This calculated result was close to the simulation result. However, when compared to the thickness under a zero electrical field, this small thickness variation is negligible. In addition, since the PVDF sheet was embedded with metalized electrodes on both sides, the surface area of the metalized electrodes did not change. This implies that the variation in thickness and surface area for this material can be neglected for this specific PVDF material packaging. Thus, the capacitance change of the capacitor only depended on the permittivity change. 

### 2.4. Experimental Measurement

**Figure 4:**
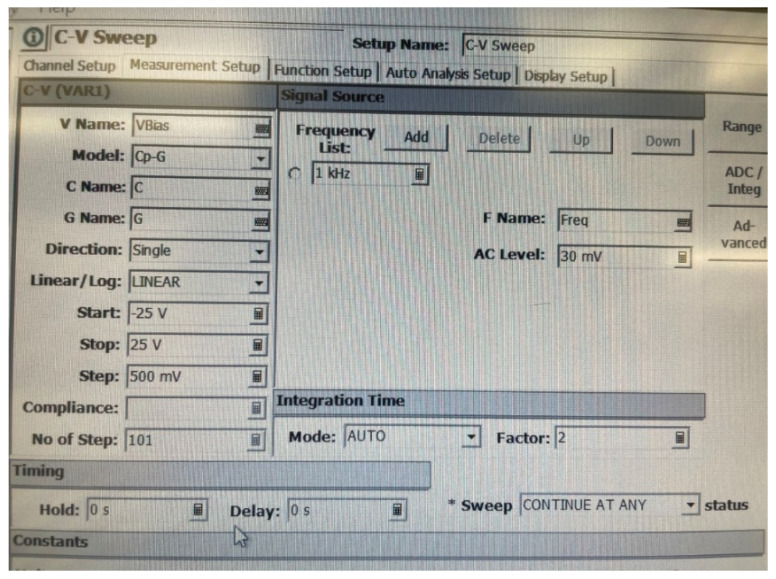
Measurement settings for the B1500A Semiconductor Device Analyzer.

The PVDF sheet used in the experiment had the same dimensions as in the simulation. The Agilent Technologies B1500A Semiconductor Device Analyzer was used to obtain the C-V curve of the piezoelectric PVDF in this work. The measurement settings used for this machine are shown in Figure 4. The maximum DC output voltage this machine can generate is 25 V and the minimum voltage is −25 V, so the start and stop DC voltages were −25 V and 25 V while the voltage increase step was 500 mV. To measure the capacitance, a 30 mV AC voltage with a frequency of 1 kHz was used. The C-V curve was recorded while sweeping DC voltages from −25 V to +25 V. The same measurement settings were used to measure the capacitance change under different DC voltages at different temperatures of 25, 40, 50, 60, and 70 °C.

## 3. Results

The results include the capacitance of the VS applied voltage and permittivity of the VS electrical field. The capacitance of the VS applied voltage was obtained with the Agilent Semiconductor Device Analyzer. The thickness and surface area were constant. The variation in capacitance was due to variations in permittivity. According to Equation (5), the value of permittivity as the capacitance changes can be calculated. 

**Figure 5:**
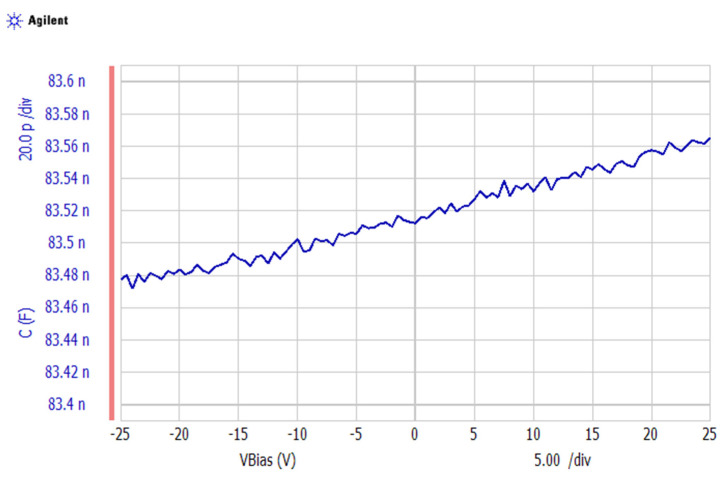
Measured C-V curve when applying voltages ranging from −25 V to +25 V at room temperature (25 °C).

The C-V curve obtained from the B1500A Semiconductor Device Analyzer at room temperature (25 °C) is shown in Figure 5. The applied voltage ranged from −25 V to +25 V. When the voltage increased, the measured capacitance of the PVDF sheet increased. 

**Figure 6:**
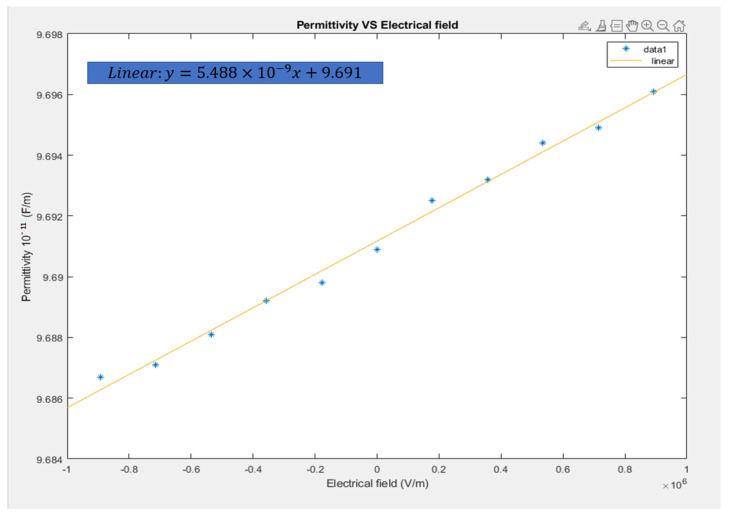
Permittivity vs. Electrical field at room temperature (25 °C).

The permittivity of this PVDF sheet was determined based on  ε = C × Th/A. The applied voltage divided by the thickness was used to determine the electrical field based on *E* = Applied voltage/Th. The area and thickness were unchangeable in the equations when different voltages were applied. Thus, as shown in Figure 5, the electrical field could be used to replace voltage/Th. Thus, the permittivity was taken as C × Th/A. Figure 6 plots the permittivity vs. the electrical field with linear fitting. From this figure, we can see that the permittivity of the PVDF sheet increased from 9.6867 × 10^−11^ to 9.6961 × 10^−11^ F/m as the electrical field increased from −0.89 × 10^6^ to 0.89 × 10^6^ V/m.

**Figure 7:**
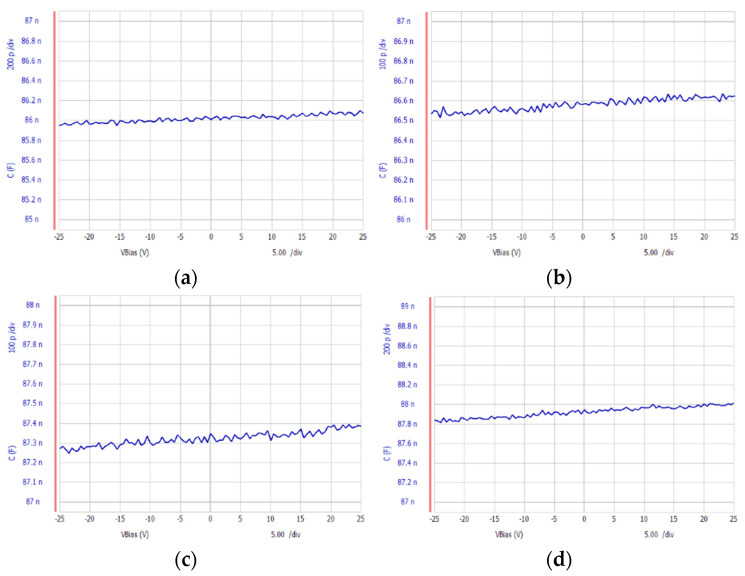
Capacitance vs. voltage at temperatures of (**a**) 40 °C, (**b**) 50 °C, (**c**) 60 °C, (**d**) 70 °C.

We also measured the capacitance change under bias voltages at different temperatures. The PVDF sheet was placed on a temperature controllable plot. At the same time, we used the Agilent Technologies B1500A Semiconductor Device Analyzer to measure the capacitance under bias voltages from −25 V to 25 V at different temperatures. Capacitance vs. Bias Voltage at different temperatures is shown in Figure 7. The capacitance increased not only when temperature increased, but also as the bias voltage increased. At room temperature (25 °C), the permittivity with zero electrical field was 9.6909 × 10^−11^ (F/m), and it ranged from 9.6867 × 10^−11^ to 9.6961 × 10^−11^ F/m under bias voltages from −25 V to 25 V. At 40 °C, the permittivity with zero electrical field was 9.9793 × 10^−11^ (F/m), and it ranged from 9.977 × 10^−11^ to 9.9886 × 10^−11^ F/m under bias voltages from −25 V to 25 V. At 50 °C, the permittivity at zero electrical field is 10.048 × 10^−11^ (F/m) and it ranges from 10.038 × 10^−11^ F/m to 10.051 × 10^−11^ F/m under the bias voltage from −25 V to 25 V. At 60 °C, the permittivity with zero electrical field was 10.135 × 10^−11^ (F/m), and it ranged from 10.127 × 10^−11^ to 10.142 × 10^−11^ F/m under bias voltages from −25 V to 25 V. At 70 °C, the permittivity with zero electrical field was 10.197 × 10^−11^ (F/m), and it ranged from 10.19 × 10^−11^ to 10.211 × 10^−11^ F/m under bias voltages from −25 V to 25 V.

**Figure 8:**
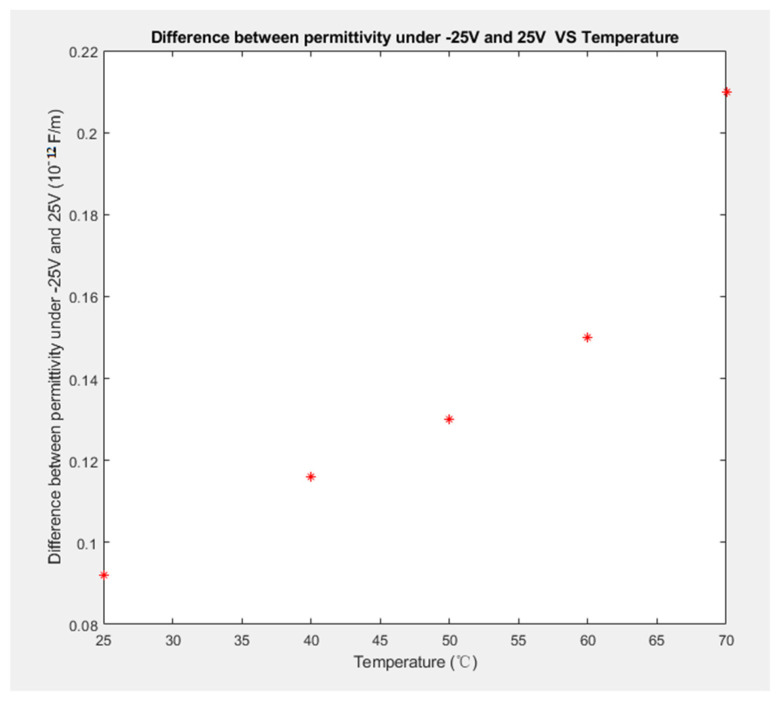
Difference in permittivity under applied voltages of −25 V and 25 V vs. Temperature.

The difference in permittivity under an applied voltage of −25 V versus the permittivity under an applied voltage of 25 V at 25, 40, 50, 60, and 70 °C was 0.0092 × 10^−11^, 0.0116 × 10^−11^, 0.013 × 10^−11^, 0.015 × 10^−11^, and 0.021 × 10^−11^ F/m, respectively, as shown in Figure 8. 

From the above data, we conclude that the electrical field can cause increases in the permittivity change at high temperatures. However, since most PVDF-based biomedical devices work at body temperature or room temperature, it is more important to know the permittivity change under an electrical field at a fixed temperature. All measurements were repeated under the same applied conditions, and the same results were obtained. 

## 4. Discussion

To the best of our knowledge, this is the first study to measure the permittivity of PVDF under the influence of an electrical field. By sweeping the voltage from negative to positive values, the capacitance change was observed through measurement with a Semiconductor Device Analyzer. The thickness change was obtained by COMSOL simulation, although the change was negligible. The area did not change due to the presence of metalized electrodes on both sides. Thus, the value of permittivity could be calculated from these results using the equation ε = C × Th/A. This proposed method for calculating permittivity does not require high resolution displacement measurement instruments, since the variations in geometric dimensions were obtained from simulation and the simulation proved that the thickness change is ignorable. In addition, the metalized electrodes on both sides kept the capacitor area nearly unchangeable. For the material sample used here, there were very small variations in the thickness and area of the sample. However, we obtained large variations in the permittivity of the material in the range from 9.6867 × 10^−11^ to 9.6961 × 10^−11^ F/m. In addition, we measured the permittivity change by applying an electrical field at different temperatures. The results show that at high temperatures, the electrical field caused more variation in permittivity. However, most PVDF-based biomedical devices are wearable at room temperature or implanted at body temperature, which does not change a lot. Thus, the permittivity change under an electrical field is more important. Further studies will investigate changes in piezoelectric coefficients of PVDF and other materials under different electrical fields and temperatures. 

## Data Availability

Publicly available datasets were analyzed in this study. This data can be found here: https://www.cdiweb.com/products/detail/1-1004347-0/185115/.

## References

[1] Liu C. (2006). Piezoelectric Sensing and Actuation. Foundations of MEMS.

[2] Dong B., Zaghloul M. (2019). Generation and enhancement of surface acoustic waves on a highly doped p-type GaAs substrate. Nanoscale Adv..

[3] Sukesha Vig R., Kumar N. (2015). Effect of Electric Field and Temperature on Dielectric Constant and Piezoelectric Constant of Piezoelectric Materials: A Review. Integr. Ferroelectr..

[4] Dong B., Afanasev A., Johnson R., Zaghloul M. (2020). Enhancement of Photoemission on p-Type GaAs Using Surface Acoustic Waves. Sensors.

[5] Hinchet R., Khan U., Falconi C., Kim S.W. (2018). Piezoelectric properties in two-dimensional materials: Simulations and experiments. Mater. Today.

[6] Zhou Y. (2020). Design and Simulation of SAW-Driven Drug Delivery Device. Master’s Thesis.

[7] Sukesha R., Kumar N. Variation of piezoelectric coefficient and dielectric constant with electric field and temperature: A review. Proceedings of the 2014 Recent Advances in Engineering and Computational Sciences (RAECS).

[8] Park W., Yang J.S., Kang C.G., Lee Y.S., Hwang H.J., Cho C., Lim S.K., Kang S.C., Hong W.K., Lee S.K. (2013). Characteristics of a pressure sensitive touch sensor using a piezoelectric PVDF-TrFE/MoS2 stack. Nanotechnology.

[9] Abbasipour M., Khajavi R., Yousefi A.A., Yazdanshenas M.E., Razaghian F. (2017). The piezoelectric response of electrospun PVDF nanofibers with graphene oxide, graphene, and halloysite nanofillers: A comparative study. J. Mater. Sci. Mater. Electron..

[10] Wang S., Dong B., Zaghloul M. Interface Electronics Design for Wireless Generation of Surface Acoustic Wave Utilized in Wearable Drug Delivery Application. Proceedings of the 2020 XXXIIIrd General Assembly and Scientific Symposium of the International Union of Radio Science (URSI GASS).

[11] Rajala S., Lekkala J. (2012). Film-Type Sensor Materials PVDF and EMFi in Measurement of Cardiorespiratory Signals—A Review. IEEE Sens. J..

[12] Jacobs J.L., Embree P., Glei M., Christensen S., Sullivan P.K. Characterization of a novel heart and respiratory rate sensor. Proceedings of The 26th Annual International Conference of the IEEE Engineering in Medicine and Biology Society.

[13] Choi S., Jiang Z. (2006). A novel wearable sensor device with conductive fabric and PVDF film for monitoring cardiorespiratory signals. Sens. Actuators A Phys..

[14] Ansourian M.N., Dripps J.H., Jordan J.R., Beattie G.J., Boddy K. (1993). A transducer for detecting foetal breathing movements using PVDF film. Physiol. Meas..

[15] Chen W., Zhu X., Nemoto T., Kanemitsu Y., Kitamura K., Yamakoshi K. (2005). Unconstrained detection of respiration rhythm and pulse rate with one under-pillow sensor during sleep. Med. Biol. Eng. Comput..

[16] Niizeki K., Nishidate I., Uchida K., Kuwahara M. (2005). Unconstrained cardiorespiratory and body movement monitoring system for home care. Med. Biol. Eng. Comput..

[17] Bai P., Wang S., Jia J., Wang H., Yang W. (2021). Effect of BaTiO3 nanowire on effective permittivity of the PVDF composites. AIP Adv..

[18] Piezoelectric Materials: Understanding the Standards. https://www.comsol.com/blogs/piezoelectric-materials-understanding-standards/.

[19] Ahmad M.A., Allataifeh A. (2018). Electrical extraction of piezoelectric constants. Heliyon.

[20] Piezo Film Sheets from TE Connectivity. https://www.cdiweb.com/portalproductdetail.aspx?ProdId=185115&ManufId=355.

